# Going Beyond Antiarrhythmic–Anticoagulant Drug Interactions: Considerations in the Complex Medicated Patient

**DOI:** 10.19102/icrm.2019.100301

**Published:** 2019-03-15

**Authors:** James A. Reiffel

**Affiliations:** ^1^Department of Medicine, Division of Cardiology, Electrophysiology Section, Columbia University, New York, NY, USA

**Keywords:** Antiarrhythmic, anticoagulation, drug–drug interaction

Interactions are a part of life. They may be social, professional, athletic, or more. Notably, each such interaction can have an impact on some aspect of our life. So too it is with drug–drug interactions—both pharmacokinetic and pharmacodynamic in nature—which can be beneficial, harmful, or innocuous. In this issue of *The Journal of Innovations in Cardiac Rhythm Management*, Drs. Kaja Konieczny and Paul Dorian explore the numerous potential and documented drug–drug interactions between antiarrhythmic drugs (AADs) and oral anticoagulants (OACs), many of which have important clinical consequences.^1^ In this commentary to supplement their paper, I would like to highlight some of the major points they made and add a few more.

After briefly discussing some basic but important principles of drug interactions, Konieczny et al. reviewed in concise but remarkable detail the metabolic and clearance pathways for each of our available AADs and OACs to a degree worthy of many pharmacologic textbooks, yet also to a point that is quite suitable for busy clinicians. Their tables should be particularly useful to practitioners considering the use of an AAD in the setting of an OAC or vice versa. Importantly, although dabigatran, rivaroxaban, apixaban, and edoxaban all have renal clearance via a p-glycoprotein (P-gp) pathway, the three latter factor Xa inhibitors also boast clearance via hepatic metabolism. Accordingly, although P-gp inhibitors will raise the levels of dabigatran and force consideration of a dose reduction, the same is not true for the factor Xa inhibitors unless there is a simultaneous inhibition of hepatic metabolism, as, without the latter, the liver can handle clearance of the portion of the drug no longer cleared by the kidney. Understanding this concept will help to explain some of the interactions that the authors discuss, such as those in their **Table 3**, and may help clinicians to better consider potential actions when faced with the need to make drug-related decisions where no published data on the interactions yet exist.

Notably, however, not discussed in and not the purpose of the aforementioned paper are several additional drug–drug interactions relevant to AADs and OACs for which important interactions exist, but not between each other. In the care of patients, physicians must consider interactions with agents that are neither AADs nor OACs but which interact with them via the same clearance mechanisms as are discussed in the paper in question. For example, the package inserts (usually sections 7 and/or 12) for the new direct-acting OACs (DOACs) list several drugs (including multiple antiviral, antifungal, antibiotic, antiseizure, cardioactive, and herbal agents) that can inhibit or induce their metabolism and/or renal clearance. Unfortunately, the listed agents are not identical or treated similarly in each of these package inserts. For example, the methods of handling verapamil and diltiazem, which are both P-gp and moderate cytochrome P450 (CYP) 3A4 inhibitors, are not identical across the package inserts, nor are they consistent in the package inserts for any single OAC that has been revised periodically since the original release of the drug. Thus, practitioners may well find themselves uncertain as to the appropriate response to drug dosing when such drug interactions are anticipated—especially when clinical trial data are not available to indicate any consequences or lack thereof when the combination is used. Moreover, there are many P-gp and/or hepatic metabolism–inhibiting or hepatic metabolism–inducing drugs that are not listed in the package inserts for either OACs or AADs, yet they are used in the care of our patients for their underlying cardiovascular or other systemic disorders. Although the examples are too numerous to discuss here, extensive online references do exist that clinicians can use, with one such useful online source being maintained by *The Medical Letter* (www.themedicalletter.org). When faced with the absence of clinical data on any such combination that may arise, clinicians will need to use their best judgement, as guided by the information and examples supplied in the manuscript by Konieczny et al.

Perhaps even more problematic is the circumstance of when multiple drugs are used together. Consider the fact that most patients with atrial fibrillation (AF), for whom both AADs and OACs are used, are older and have multiple comorbidities (all of which contribute to their risk for thromboembolism and their indication for an OAC). Multimorbidity, defined as two or more chronic conditions, is remarkably common. Published reports have noted multimorbidity prevalence rates of 10% to more than 60% in the older population, including more than two-thirds of Americans aged 65 years or older and more than 80% of Americans aged at least 75 years, with half of adults aged 75 years or older having four or more chronic conditions and 20% having six or more.^2–5^ In the pivotal Rivaroxaban Once-daily Oral Direct Factor Xa Inhibition Compared with Vitamin K Antagonism for Prevention of Stroke and Embolism Trial in Atrial Fibrillation (ROCKET AF) study^6^ in AF patients, the average number of drugs used by a patient was nine! Most patients with hypertension take two to three antihypertensives alone. Similarly, most patients with diabetes take multiple agents just for this single disorder. Then, consider the situation if the AAD chosen is dofetilide, for which a long list of drug interactions exists, or is amiodarone, which interacts in a metabolic inhibitory manner with almost every drug that has been tested with it. How is a practitioner then expected to make a truly data-based decision when initiating an OAC? Dofetilide is metabolized by CYP3A4 and excreted by the renal cation transport system, independent of P-gp. Among the many agents with which dofetilide interacts are verapamil (contraindicated for use with dofetilide) and diltiazem, both of which are often used for rate control in AF. Diltiazem is an inhibitor of both P-gp and CYP3A4 and should also have interactions with the new OACs, but this has not yet been well-tested. Drugs that inhibit CYP3A4 and/or the renal transport system (such as triamterene, not uncommonly used in hypertensives) may also interact with dofetilide. Consider then a patient who is treated with both diltiazem and rivaroxaban, and now add some degree of renal impairment and multiple antihypertensive agents. Choosing the most appropriate dose of rivaroxaban could be both complex and uncertain. The situation could be even worse with amiodarone, which is mainly metabolized by CYP3A4; is a potent inhibitor of the CYPs 1A2, 2C9, 2D6, and 3A4; and additionally may interact with other drugs (such as digoxin) via the inhibition of the P-gp membrane transporter system. Yet, as complex as this all may be, post-hoc analyses of several of the DOAC versus warfarin trials as well as other reports have not consistently revealed increased bleeding events in patients on DOACs who have also taken amiodarone.^7–10^ This contrasts with findings in older trials in which the long-recognized amiodarone–warfarin interaction has resulted in markedly elevated international normalized ratio values and a need to alter warfarin dosing. Importantly, these inconsistent observations pose a clinical dilemma for practitioners. Again, an adequate review of package inserts, an understanding of the pharmacologic principles discussed by Konieczny et al., the consideration of any clinical trial data that exist, and the incorporation of personal experience are all necessary and should collectively serve as the basis of therapeutic dosing decisions **(Figure 1)**.

Finally, two additional AAD interactions are worth noting, although they are not linked to OACs. The first is the use of two or more AADs in combination. This may be done to enhance efficacy by employing multiple antiarrhythmic electrophysiologic actions and/or to improve tolerance.^11–13^ Examples of the former include quinidine plus mexiletine to better control ventricular arrhythmias or amiodarone plus any of several AADs used in combination with it for both ventricular and atrial tachyarrhythmias. An example of the latter is the combination of quinidine plus disopyramide, each in reduced dose form, not only to maintain antiarrhythmic efficacy but also to offset and reduce gastrointestinal intolerance. With respect to amiodarone, it should always be recalled that it has pharmacokinetic and often pharmacodynamic interactions with drugs with which it has been studied in combination with; hence, when adding another AAD to amiodarone, a good rule is to begin with half of the usual starting dose. The second additional AAD interaction to note is that of drug–device interactions.^14–16^ AADs can alter the threshold current needed for capture by pacemakers or that needed for defibrillation. In general, sodium channel blockers increase both of these values, while pure potassium channel blockers decrease them or leave them unchanged. There certainly are exceptions to this general rule, with some interspecies differences, but, nonetheless, these are additional interactions appropriate to keep in mind when starting, stopping, or changing an AAD regimen.

To conclude, the use of AADs and OACs is complex enough on its own. However, when used in combination, especially in the complex patients that require them and who may have multiple comorbid disorders and polypharmacy treatment regimens, the use of clinical judgment and outstanding resources such as that provided by Konieczny et al. is essential. Hopefully, the desired outcomes will be safely and effectively attained.

## Figures and Tables

**Figure 1:**
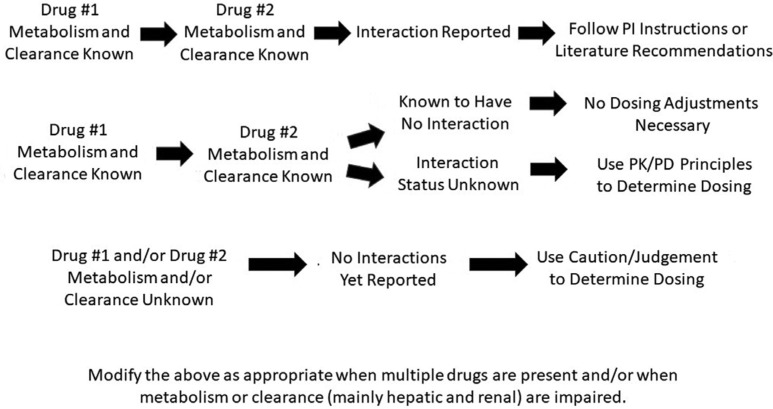
Drug dosing based upon drug pharmacology and drug interaction data. PI: package insert; PK: pharmacokinetic; PD: pharmacodynamic.
